# Predicting lymph node metastasis in colorectal cancer patients: development and validation of a column chart model

**DOI:** 10.1007/s13304-024-01884-6

**Published:** 2024-07-02

**Authors:** Xiaoqiang Niu, Jiaqing Cao

**Affiliations:** Department of Gastrointestinal Surgery, The Second Affiliated Hospital, Jiangxi Medical College, Nanchang University, Nanchang, China

**Keywords:** Colorectal cancer, Lymphatic metastasis, Nomogram, SEER, Treatment programs

## Abstract

Lymph node metastasis (LNM) is one of the crucial factors in determining the optimal treatment approach for colorectal cancer. The objective of this study was to establish and validate a column chart for predicting LNM in colon cancer patients. We extracted a total of 83,430 cases of colon cancer from the Surveillance, Epidemiology, and End Results (SEER) database, spanning the years 2010–2017. These cases were divided into a training group and a testing group in a 7:3 ratio. An additional 8545 patients from the years 2018–2019 were used for external validation. Univariate and multivariate logistic regression models were employed in the training set to identify predictive factors. Models were developed using logistic regression, LASSO regression, ridge regression, and elastic net regression algorithms. Model performance was quantified by calculating the area under the ROC curve (AUC) and its corresponding 95% confidence interval. The results demonstrated that tumor location, grade, age, tumor size, T stage, race, and CEA were independent predictors of LNM in CRC patients. The logistic regression model yielded an AUC of 0.708 (0.7038–0.7122), outperforming ridge regression and achieving similar AUC values as LASSO regression and elastic net regression. Based on the logistic regression algorithm, we constructed a column chart for predicting LNM in CRC patients. Further subgroup analysis based on gender, age, and grade indicated that the logistic prediction model exhibited good adaptability across all subgroups. Our column chart displayed excellent predictive capability and serves as a useful tool for clinicians in predicting LNM in colorectal cancer patients.

## Introduction

Colorectal cancer (CRC) refers to malignant tumors occurring in the proximal colon, distal colon, or rectum [1]. Currently, the standard treatment for CRC is curative surgery, although endoscopic resection may be considered for some early-stage colon cancer patients. However, if lymph node metastasis (LNM) is present, the treatment principles may significantly differ. For early-stage CRC with LNM, endoscopic treatment may not be suitable [2, 3]. On the other hand, neoadjuvant chemotherapy should be considered for advanced-stage CRC with LNM. Therefore, regardless of whether endoscopic or surgical resection is performed, preoperative assessment of LNM in CRC patients is essential. Predicting the presence of LNM in CRC patients before surgery holds great significance, both in treatment selection and prognostic evaluation [4]. Several studies have developed predictive models for LNM in CRC patients; however, these studies have limitations such as small sample sizes, single-center designs, or lack of external validation cohorts [5–7].

Currently, the risk factors for LNM remain unclear. To address this, we extracted data from the Surveillance, Epidemiology, and End Results (SEER) database for patients diagnosed with CRC between 2010 and 2019. Subsequently, we constructed a nomogram to predict LNM in CRC patients and evaluated the applicability of the model through external validation.

## Methods

### Study design and population

The data were extracted from the SEER database, a population-based clinical data repository proposed by the National Cancer Institute, covering approximately 28% of the U.S. population [8]. Using SEER Stat software (Calverton, Maryland), we obtained a list of cases diagnosed with CRC between 2010 and 2019 from the SEER database. Since patient data in the SEER database are publicly available and de-identified, this study was exempt from ethical review.

### Inclusion and exclusion criteria

Inclusion criteria: (1) Primary tumor located in the colon or rectum, (2) Pathological type classified as adenocarcinoma, (3) Patients with complete and available clinical baseline data. Exclusion criteria: (1) Multiple primary malignant tumors, (2) Distant metastasis present, (3) Zero survival time, (4) Diagnosed based on death certificates or autopsy reports.

### Variable categorization

We extracted data on patients' race, age (< 60 years, 60–70 years, > 70 years), gender, marital status, T stage, tumor location, tumor size (< 7 cm, 7–15 cm, > 15 cm), and CEA levels. LNM served as the endpoint indicator.

### Statistical analysis

Data were retrieved from the SEER database using SEER Stat software (version 8.4.2). The data from 2010 to 2017 were divided into training and testing sets in a 7:3 ratio. Data from 2018 to 2019 were used for external validation. Categorical variables were presented as numbers and percentages, and intergroup comparisons were performed using the chi-square test (χ^2^) or Fisher's exact test.

Univariate and multivariate logistic regression analyses were sequentially conducted to identify independent risk factors for LNM and establish a predictive model. Models were developed using logistic regression, LASSO regression, ridge regression, and elastic net regression methods. The Hosmer–Lemeshow goodness-of-fit test was performed to assess the fitness of the predictive model. Model performance was quantified by calculating the area under the receiver operating characteristic curve (AUC) with its corresponding 95% confidence interval (CI), sensitivity, specificity, positive predictive value (PPV), and negative predictive value (NPV). The optimal-performing model was selected, and a nomogram was constructed.

All statistical analyses and visualizations were performed using R software (version 4.3.1). The glmnet package was used for constructing LASSO regression, ridge regression, and elastic net regression models with ten-fold cross-validation. Other R packages, such as compareGroups, ResourceSelection, rms, and pROC, were also utilized. A p value < 0.05 was considered statistically significant.

## Result

### Baseline characteristics

A total of 83,430 cases from the SEER database were included in this study as the training and testing sets for the period between 2010 and 2017, and 18,545 cases from 2018 to 2019 were used as the external validation set (Table 1). Among these 83,430 patients, 43,889 (52.6%) were male. The racial distribution consisted of 65,641 (78.7%) White individuals, 8,776 (10.5%) Black individuals, and 9,013 (10.8%) individuals from other ethnicities. From Table 1, it can be observed that none of the univariate variables showed statistical significance between the testing and training sets. More detailed features are presented in Table 1.

**Table 1:** Characteristics of all patients

	Test (N = 25,030)	Overall (N = 83,430)	X-squared	p-value
*Age*			0.951	0.622
< 60	7614 (30.4%)	25,536 (30.6%)		
60–70	7170 (28.6%)	23,727 (28.4%)		
> 70	10,246 (40.9%)	34,167 (41.0%)		
*Sex*			0.072	0.789
Female	11,881 (47.5%)	39,541 (47.4%)		
Male	13,149 (52.5%)	43,889 (52.6%)		
*Marital status*			0.496	0.481
Married	13,546 (54.1%)	45,308 (54.3%)		
Other	11,484 (45.9%)	38,122 (45.7%)		
*CEA*			0.0294	0.864
CEA negative	15,876 (63.4%)	52,956 (63.5%)		
CEA positive	9154 (36.6%)	30,474 (36.5%)		
*Race*			3.276	0.194
White	19,741 (78.9%)	65,641 (78.7%)		
Black	2561 (10.2%)	8776 (10.5%)		
Other	2728 (10.9%)	9013 (10.8%)		
*Grade*			6.645	0.084
Grade I	1992 (8.0%)	6433 (7.7%)		
Grade II	19,071 (76.2%)	63,466 (76.1%)		
Grade III	3350 (13.4%)	11,370 (13.6%)		
Grade IV	617 (2.5%)	2161 (2.6%)		
*T_stage*			3.561	0.313
T1	3059 (12.2%)	9937 (11.9%)		
T2	4322 (17.3%)	14,380 (17.2%)		
T3	14,323 (57.2%)	47,940 (57.5%)		
T4	3326 (13.3%)	11,173 (13.4%)		
*Tumor_Site*			0.722	0.396
Colon Cancer	20,094 (80.3%)	66,826 (80.1%)		
Rectal Cancer	4936 (19.7%)	16,604 (19.9%)		
*Tumor_size*			2.541	0.281
< 7 cm	648 (2.6%)	2225 (2.7%)		
7-15 cm	1741 (7.0%)	5654 (6.8%)		
> 15 cm	22,641 (90.5%)	75,551 (90.6%)		
*LNM*			0.351	0.554
No	14,907 (59.6%)	49,558 (59.4%)		
Yes	10,123 (40.4%)	33,872 (40.6%)		

*LNM* lymph node metastasis.

### Differences in characteristics between patients with and without LNM

Table 2 displays the characteristics of patients with and without LNM. The results indicate significant differences between patients with and without LNM in terms of age, tumor location, T stage, tumor size, tumor grade, CEA levels, and race (all P < 0.001). The occurrence rate of LNM was higher in patients younger than 60 years compared to other age groups (P < 0.001). CEA-positive patients had a higher LNM occurrence rate (P < 0.001). As tumor grade increased, the proportion of LNM also increased (P < 0.001). LNM in rectal cancer was significantly higher than in colon cancer. Additionally, the occurrence rate of LNM increased with larger tumor diameters.

**Table 2:** Difference analysis of patients with or without LNM in the training set

	No (N = 34,651)	Yes (N = 23,749)	Overall (N = 58,400)	X-squared	p-value
*Age*				730.31	< 0.01
< 60	9294 (26.8%)	8628 (36.3%)	17,922 (30.7%)		
60–70	9815 (28.3%)	6742 (28.4%)	16,557 (28.4%)		
> 70	15,542 (44.9%)	8379 (35.3%)	23,921 (41.0%)		
*Sex*				0.405	0.524
Female	16,450 (47.5%)	11,210 (47.2%)	27,660 (47.4%)		
Male	18,201 (52.5%)	12,539 (52.8%)	30,740 (52.6%)		
*Marital status*				1.93	0.1648
Married	18,763 (54.1%)	12,999 (54.7%)	31,762 (54.4%)		
Other	15,888 (45.9%)	10,750 (45.3%)	26,638 (45.6%)		
*CEA*				823.94	< 0.01
CEA negative	23,642 (68.2%)	13,438 (56.6%)	37,080 (63.5%)		
CEA positive	11,009 (31.8%)	10,311 (43.4%)	21,320 (36.5%)		
*Race*				91.255	< 0.01
White	27,682 (79.9%)	18,218 (76.7%)	45,900 (78.6%)		
Black	3536 (10.2%)	2679 (11.3%)	6215 (10.6%)		
Other	3433 (9.9%)	2852 (12.0%)	6285 (10.8%)		
*Grade*				1245.1	< 0.01
Grade I	3209 (9.3%)	1232 (5.2%)	4441 (7.6%)		
Grade II	27,185 (78.5%)	17,210 (72.5%)	44,395 (76.0%)		
Grade III	3579 (10.3%)	4441 (18.7%)	8020 (13.7%)		
Grade IV	678 (2.0%)	866 (3.6%)	1544 (2.6%)		
*T_stage*				5660.5	< 0.01
T1	6022 (17.4%)	856 (3.6%)	6878 (11.8%)		
T2	7838 (22.6%)	2220 (9.3%)	10,058 (17.2%)		
T3	17,730 (51.2%)	15,887 (66.9%)	33,617 (57.6%)		
T4	3061 (8.8%)	4786 (20.2%)	7847 (13.4%)		
*Tumor_Site*				154.3	< 0.01
Colon Cancer	28,318 (81.7%)	18,414 (77.5%)	46,732 (80.0%)		
Rectal Cancer	6333 (18.3%)	5335 (22.5%)	11,668 (20.0%)		
*Tumor_size*				1265.5	< 0.01
< 7 cm	1364 (3.9%)	213 (0.9%)	1577 (2.7%)		
7-15 cm	3115 (9.0%)	798 (3.4%)	3913 (6.7%)		
> 15 cm	30,172 (87.1%)	22,738 (95.7%)	52,910 (90.6%)		

*LNM*: lymph node metastasis

### Risk factors associated with LNM in CRC patients

The results of univariate and multivariate logistic regression analyses are presented in Table 3. Multivariate logistic regression analysis revealed that T stage, CEA levels, tumor size, and tumor grade were independent risk factors for LNM in CRC patients. The risk of LNM in rectal cancer patients was 1.4 times higher than in colon cancer patients (OR 1.40; 95% CI 1.34–1.46). Compared to patients with tumor diameter < 7 cm, the risk of LNM occurrence in patients with tumor diameters of 7–15 cm and > 15 cm was 1.20 times (OR 1.20; 95% CI 1.01–1.43) and 1.36 times (OR 1.36; 95% CI 1.16–1.61), respectively. Compared to patients with grade I tumors, the risk of LNM occurrence in patients with grade II, III, and IV tumors was 1.31 times (OR 1.31; 95% CI 1.21–1.41), 2.32 times (OR 2.32; 95% CI 2.13–2.52), and 2.39 times (OR 2.39; 95% CI 2.11–2.72), respectively. Compared to T1 stage, the risk of LNM occurrence in patients with T2, T3, and T4 stages was 1.18 times (OR 1.18; 95% CI 1.68–2.03), 5.40 times (OR 5.40; 95% CI 4.96–5.89), and 8.89 times (OR 8.89; 95% CI 8.08–9.80), respectively. White individuals had the lowest risk of LNM compared to Black individuals and individuals from other races. CEA-positive patients had a 1.28 times higher LNM occurrence rate compared to CEA-negative patients (OR 1.28; 95% CI 1.24–1.33). The occurrence rates of LNM in patients aged 60–70 years and > 70 years were 0.76 times (OR 0.76; 95% CI 0.73–0.80) and 0.55 times (OR 0.55; 95% CI 0.53–0.58), respectively, compared to patients younger than 60 years.

**Table 3:** Univariate and multivariate logistic regression analyses of factors associated with LNM

Variables	Univariate analysis		Multivariate analysis	
	OR (95% CI)	*P*	OR (95% CI)	*P*
*Sex*				
Female	Ref.			
Male	1.011 (0.978–1.045)	0.519		
*Tumor_Site*				
Colon Cancer	Ref.			
Rectal Cancer	1.296 (1.243–1.350)	< 0.01	1.402 (1.340–1.466)	< 0.001
*Grade*				
Grade I	Ref.			
Grade II	1.649 (1.540–1.766)	< 0.01	1.307 (1.215–1.407)	< 0.001
Grade III	3.232 (2.987–3.499)	< 0.01	2.316 (2.127–2.521)	< 0.001
Grade IV	3.327 (2.951–3.752)	< 0.01	2.393 (2.108–2.719)	< 0.001
*Tumor_size*				
< 7	Ref.			
7–15	1.641 (1.395–1.937)	< 0.01	1.200 (1.01–1.430)	0.040236
> 15	4.826 (4.183–5.596)	< 0.01	1.363 (1.160–1.606)	< 0.001
*T_stage*				
T1	Ref.			
T2	1.993 (1.830–2.172)	< 0.01	1.846 (1.682–2.028)	< 0.001
T3	6.304 (5.853–6.797)	< 0.01	5.399 (4.956–5.888)	< 0.001
T4	11.000 (10.111–11.978)	< 0.01	8.891 (8.078–9.796)	< 0.001
*Race*				
White	Ref.			
Black	1.151 (1.091–1.215)	< 0.01	1.107 (1.04–1.172)	< 0.001
Other	1.262 (1.197–1.331)	< 0.01	1.215 (1.148 1.286)	< 0.001
*Age*				
< 60	ref			
60–70	0.740 (0.709–0.772)	< 0.01	0.763 (0.729–0.799)	< 0.001
> 70	0.581 (0.558–0.604)	< 0.01	0.551 (0.528–0.575)	< 0.001
*CEA*				
CEA negative	Ref.			
CEA positive	1.648 (1.592–1.705)	< 0.01	1.282 (1.235–1.330)	< 0.001
*Marital status*				
Married	Ref.			
Other	0.977 (0.945–1.010)	0.162		

*LNM* lymph node metastasis, *OR* odds ratio, *CI* confidence interval, *Ref* reference

### Model comparison and selection

**Fig. 1:**
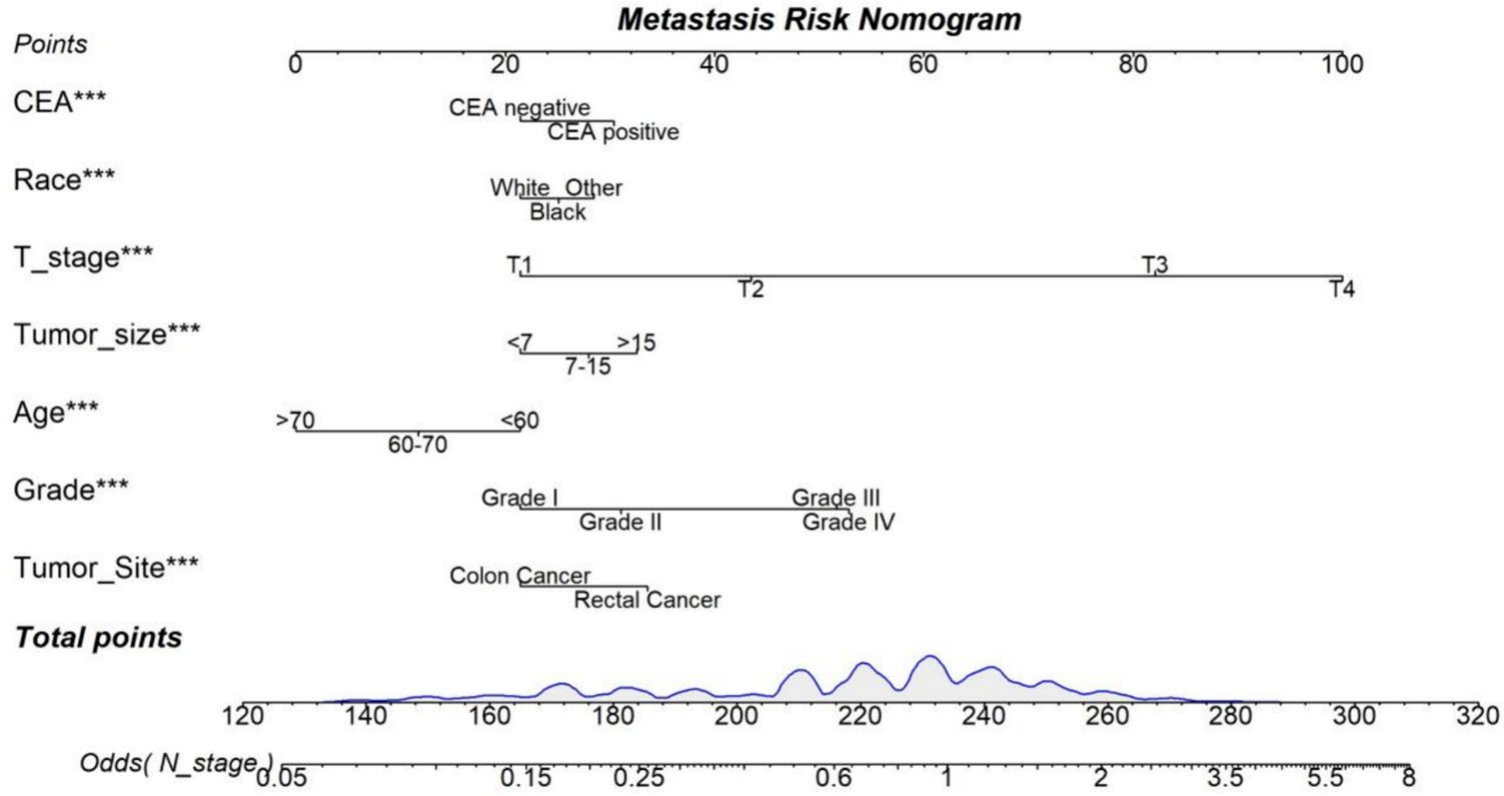
Nomogram for predicting lymph node metastasis (LNM) in colorectal cancer (CRC) patients

We constructed models using logistic regression, LASSO regression, ridge regression, and elastic net regression. Table 4 presents the AUC values of these models in the training and testing sets. In the testing set, the logistic regression, LASSO regression, ridge regression, and elastic net regression models achieved AUCs of 0.708 (95% CI 0.704–0.712), 0.707 (95% CI 0.703–0.711), 0.708 (95% CI 0.704–0.712), and 0.708 (95% CI 0.702–0.714), respectively. There were no significant differences in AUC among these models (P > 0.05). Although all models performed similarly, the logistic regression model was more clinically interpretable. Therefore, the logistic regression model was selected (Fig. 1).

**Table 4:** The area under the receiver operating characteristic curve (AUC) for different models

Models	Training set	Test set
	AUC (95% CI)	AUC (95% CI)
Logistic regression	0.708 (0.704–0.712)	0.708 (0.701–0.714)
Ridge regression	0.707 (0.703–0.711)	0.707 (0.701–0.714)
LASSO regression	0.708 (0.704–0.712)	0.708 (0.701–0.714)
Elastic-net regression	0.708 (0.702–0.714)	0.708 (0.702–0.714)

Nomogram for prediction of LNM in EGC patients

### Nomogram for predicting LNM in CRC patients

**Fig. 2:**
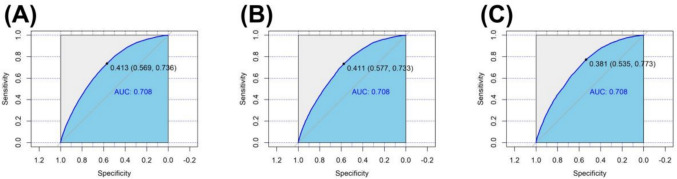
Receiver operator characteristic (ROC) curves and the area under the ROC curve (AUC) for the logistic regression prediction model in the training set, test set, and external validation. **A** ROC curves in the training set; **B** ROC curves in the test set; **C** ROC curves in the external validation

Table 5 displays the performance of the logistic regression model. In the testing set, the logistic regression model achieved an AUC of 0.708 (95% CI 0.704–0.712), accuracy of 0.637 (95% CI 0.63–0.641), sensitivity of 0.736 (95% CI 0.730–0.742), specificity of 0.569 (95% CI 0.564–0.574), PPV of 0.539 (95% CI 0.534–0.545), and NPV of 0.759 (95% CI 0.753–0.764). The Hosmer–Lemeshow goodness-of-fit test indicated good calibration of the predictive model (χ^2^ = 10.207, P = 0.251). Furthermore, during external validation, the model achieved an AUC of 0.709 (95% CI 0.701–0.716), indicating its good applicability to external validation data (Fig. 2, Table 5).

**Table 5:** The performance of the logistic regression prediction model

Parameter (95% CI)	Training set	Test set	External validation
AUC	0.708 (0.704–0.712)	0.708 (0.702–0.714)	0.709 (0.701–0.716)
Accuracy	0.637 (0.6329–0.641)	0.640 (0.634–0.646)	0.625 (0.617–0.631)
Sensitivity	0.736 (0.7304–0.742)	0.733 (0.724–0.742)	0.773 (0.763–0.782)
Specificity	0.569 (0.564–0.574)	0.577 (0.568–0.585)	0.535 (0.526–0.543)
PPV	0.539 (0.534–0.545)	0.541 (0.532–0.549)	0.502 (0.492–0.511)
NPV	0.759 ( 0.753–0.764)	0.761 (0.753–0.770)	0.795 (0.786–0.803)
Cutoff	0.413	0.411	0.413

*AUC* the area under the receiver operating characteristic curve, *PPV* positive predictive value, *NPV* negative predictive value

### Further validation based on different subgroups

Further validation was conducted based on gender, age, and tumor grade (Table 6). In the testing set, the logistic regression predictive model exhibited good performance for male and female patients, as well as patients aged < 60 years, 60–70 years, and > 70 years, and those with grade I tumors. The AUC values for these subgroups were 0.705 (95% CI 0.696–0.714), 0.711 (95% CI 0.702–0.720), 0.685 (95% CI 0.673–0.696), 0.708 (95% CI 0.696–0.720), 0.704 (95% CI 0.694–0.714), and 0.737 (95% CI 0.714–0.760), respectively. In the external validation dataset, the predictive model also demonstrated good applicability to these subgroups, with AUC values of 0.710 (95% CI 0.700–0.720), 0.707 (95% CI 0.696–0.718), 0.696 (95% CI 0.683–0.710), 0.710 (95% CI 0.696–0.724), 0.705 (95% CI 0.694–0.717), and 0.746 (95% CI 0.720–0.771).

**Table 6:** The performance of the prediction model based on different populations

Subgroup	Parameter (95% CI)	Test set	External validation
Gender (males)	AUC	0.705 (0.696–0.714)	0.710 (0.700–0.720)
	Sensitivity	0.740 (0.729–0.751)	0.760 (0.746–0.774)
	Specificity	0.569 (0.558–0.580)	0.552 (0.539–0.564)
	PPV	0.540 (0.528–0.551)	0.513 (0.501–0.527)
	NPV	0.762 (0.751–0.773)	0.788 (0.775–0.800)
	Accuracy	0.639 (0.630–0.647)	0.632 (0.622–0.641)
Gender (females)	AUC	0.711 (0.7015–0.7199)	0.707 (0.700–0.718)
	Sensitivity	0.688 (0.675–0.702)	0.780 (0.766–0.794)
	Specificity	0.624 (0.613–0.635)	0.527 (0.515–0.541)
	PPV	0.552 (0.539–0.566)	0.495 (0.482–0.509)
	NPV	0.748 (0.737–0.759)	0.801 (0.788–0.814)
	Accuracy	0.650 (0.642–0.658)	0.621 (0.611–0.632)
Age (< 60 years)	AUC	0.685 (0.6728–0.6963)	0.696 (0.683–0.710)
	Sensitivity	0.839 (0.827–0.851)	0.633 (0.613–0.654)
	Specificity	0.424 (0.409–0.439)	0.652 (0.634- 0.669)
	PPV	0.567 (0.554–0.581)	0.584 (0.566–0.601)
	NPV	0.745 (0.726–0.763)	0.698 (0.680–0.714)
	Accuracy	0.620 (0.610–0.631)	0.644 (0.631–0.657)
Age (60–70 years)	AUC	0.708 (0.696–0.72)	0.710 (0.696–0.724)
	Sensitivity	0.681 (0.663–0.698)	0.846 (0.831–0.863)
	Specificity	0.621 (0.606–0.636)	0.474 (0.458–0.493)
	PPV	0.556 (0.540–0.572)	0.491 (0.473–0.509)
	NPV	0.736 (0.722–0.751)	0.837 (0.820–0.855)
	Accuracy	0.646 (0.635–0.657)	0.614 (0.600–0.628)
Age (> 70 years)	AUC	0.704 (0.694–0.714)	0.705 (0.694–0.717)
	Sensitivity	0.697 (0.683–0.712)	0.647 (0.628–0.665)
	Specificity	0.605 (0.594–0.617)	0.647 (0.632–0.660)
	PPV	0.485 (0.473–0.500)	0.486 (0.468–0.503)
	NPV	0.789 (0.778–0.801)	0.780 (0.767–0.792)
	Accuracy	0.637 (0.628–0.647)	0.647 (0.635–0.658)
Grade (I)	AUC	0.737 (0.714–0.760)	0.746 (0.720–0.771)
	Sensitivity	0.832 (0.801–0.863)	0.803 (0.766–0.841)
	Specificity	0.536 (0.512–0.561)	0.571 (0.545–0.597)
	PPV	0.411 (0.383–0.441)	0.346 (0.316–0.374)
	NPV	0.891 (0.871–0.910)	0.911 (0.894–0.931)
	Accuracy	0.619 (0.598–0.640)	0.622 (0.599–0.645)
Grade (II)	AUC	0.696 (0.689–0.704)	0.687 (0.678–0.696)
	Sensitivity	0.773 (0.762, 0.782)	0.756 (0.744–0.767)
	Specificity	0.521 (0.512–0.529)	0.527 (0.517–0.537)
	PPV	0.504 (0.494–0.513)	0.480 (0.470–0.492)
	NPV	0.784 (0.776–0.794)	0.788 (0.778–0.799)
	Accuracy	0.618 (0.611–0.625)	0.611 (0.603–0.618)
Grade (III)	AUC	0.665 (0.646–0.683)	0.657 (0.636–0.678)
	Sensitivity	0.632 (0.611–0.655)	0.605 (0.582–0.632)
	Specificity	0.614 (0.588–0.640)	0.628 (0.600–0.658)
	PPV	0.667 (0.646–0.688)	0.667 (0.640–0.694)
	NPV	0.577 (0.55–0.602)	0.564 (0.537–0.592)
	Accuracy	0.624 (0.608–0.640)	0.616 (0.598–0.635)
Grade (IV)	AUC	0.631 (0.586–0.676)	0.556 (0.400–0.713)
	Sensitivity	0.699 (0.651–0.748)	0.733 (0.570–0.895)
	Specificity	0.510 (0.447–0.572)	0.500 (0.318–0.692)
	PPV	0.660 (0.610–0.706)	0.629 (0.479–0.800)
	NPV	0.554 (0.487–0.615)	0.619 (0.412–0.847)
	Accuracy	0.619 (0.579–0.655)	0.625 (0.50–0.75)

AUCarea under the curve; PPVpositive predictive value; NPVnegative predictive value

### Model fit analysis

**Fig. 3:**
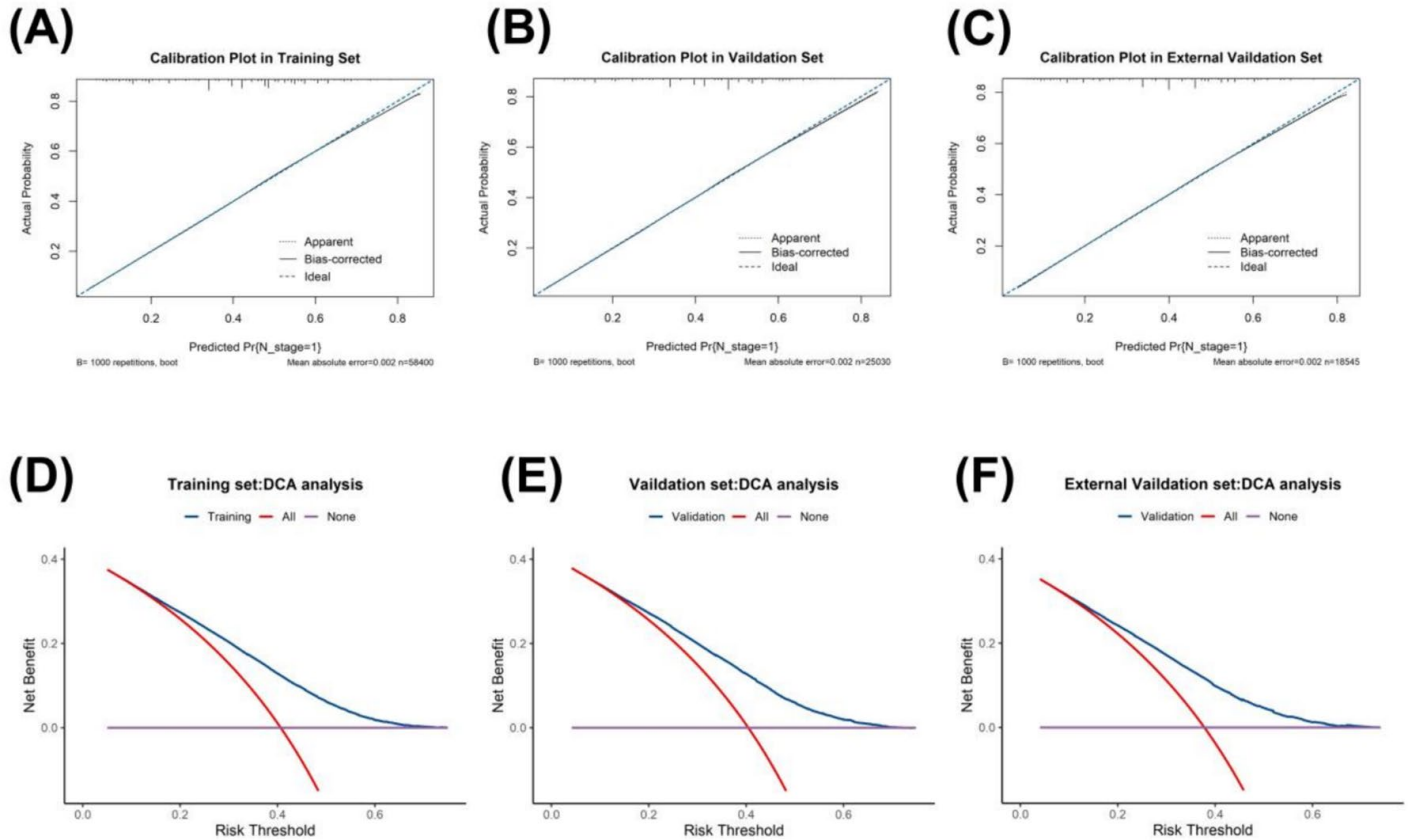
Calibration plots.: Show the consistency of the predicted potentiality and actual values。**A**–**C** The consistency of the predicted potentiality and actual values in the training set、the test set and in the external validation. **D**, **E** Decision curve analysis (DCA). Assessing clinical utility in the training set、the test set and in the external validation

The calibration curves of the nomogram (Fig. 3A–C) demonstrated high consistency between predicted and observed survival probabilities in both the training and validation cohorts. Additionally, the decision curve analysis (DCA) curves (Fig. 3D–F) indicated the good clinical utility of our model.

## Discussion

Colorectal cancer (CRC) is the third leading cause of cancer-related deaths worldwide, with over 1.85 million new cases and 850,000 deaths annually [9]. Lymph node metastasis (LNM) is an important prognostic factor in CRC and influences the selection of treatment options. Studies have shown that the 5-year survival rate for CRC patients with positive lymph nodes ranges from 30 to 60%, significantly lower than that for patients without lymph node involvement (5-year survival rate: 70–80%) [10]. Current treatment options for CRC include endoscopic resection, surgical resection, radiotherapy, chemotherapy, targeted therapy, and immunotherapy. Endoscopic treatment is mainly used for early-stage CRC but is not suitable for patients with LNM. For non-early-stage CRC patients, surgical resection is the primary consideration, but if LNM is present, preoperative adjuvant therapy needs to be evaluated [3]. Therefore, LNM is a crucial determinant in choosing the appropriate treatment approach and serves as an important prognostic factor for CRC recurrence and distant metastasis [11–13]. Predicting LNM can provide more accurate personalized treatment strategies, which is of paramount importance for CRC patients.

Numerous models have been developed to predict LNM in CRC patients; however, they have limitations and certain shortcomings [14]. In this study, we established a model based on the SEER database and conducted internal and external validations. We compared four models—logistic regression, lasso regression, ridge regression, and elastic net regression—and analyzed significant variable factors. The results indicated that LNM was associated with tumor location, grade, patient age, tumor size, T stage, race, and CEA level.

Previous studies and experience have shown that larger tumor size, deeper infiltration into the intestinal wall, and later stage are associated with a higher probability of lymph node metastasis [15, 16]. Our study revealed that compared to patients with tumor size < 7 cm, the risk of LNM increased by 1.20 times and 1.36 times in patients with tumor sizes of 7–15 cm and > 15 cm, respectively. The probability of lymph node metastasis also increased with higher T stages. Many previous studies have demonstrated the close correlation between tumor size and the risk of LNM [17], confirming tumor size as an independent prognostic factor. This may be related to the high expression of CCR7, as Yan C et al. found significantly higher CCR7 expression in tumors with LNM compared to those without, and CCR7 expression showed a positive correlation with tumor size. These findings align with our research results, indicating that as T stage advances and tumor size increases, the likelihood of lymph node metastasis, especially when reaching T4 stage or tumor diameter > 15 cm, significantly increases. Therefore, careful consideration should be given to the selection of treatment options.

Studies have shown that there are differences in the occurrence rates of lymph node metastasis (LNM) between rectal cancer and colon cancer [18, 19]. Our study indicates that the risk of LNM in rectal cancer patients is 1.4 times higher than that in colon cancer patients. This may be attributed to different tumor biology and anatomical characteristics. Therefore, it is recommended that for early-stage rectal cancer, radical resection rather than local excision seems to be a more reasonable approach, as the involvement of lymph nodes, which are more prone to metastasis in rectal cancer, may be the main cause of local recurrence after surgery. Similarly, rectal cancer patients may require adjuvant chemotherapy more often after local excision [20].

A study in the United States has demonstrated that younger CRC patients have a higher risk of lymph node positivity compared to older patients in an equal environment [21–23]. However, our study shows that among patients aged < 60 years, 60–70 years, and > 70 years, the probability of lymph node positivity decreases with increasing age, which is consistent with our research.Based on the above studies, we should exercise caution in endoscopic treatment for young early-stage CRC patients.

CEA plays a crucial role in the biological phenomena of tumor cells, including adhesion, immune response, and apoptosis [24]. Previous research and experience have shown that CEA levels are associated with lymph node positivity and prognosis in patients with CRC [25]. Our study indicates that CEA-positive patients have a 1.6 times higher likelihood of LNM compared to CEA-negative patients. This may be related to the mechanisms of CEA, as it enhances the metastatic potential of CRC through various pathways. In addition to being considered a pro-angiogenic molecule, CEA protects metastatic cells from death, alters the microenvironment of blood sinuses, promotes the expression of adhesion molecules, and enhances the survival of malignant tumor cells [26].

We developed a nomogram based on the SEER database to predict LNM in CRC patients and conducted internal and external validations. Furthermore, we further evaluated the performance of the model in different subgroups. This predictive tool for the likelihood of LNM in CRC patients can guide clinicians in selecting more appropriate treatment strategies. However, this study still has some limitations. Firstly, the data used in the study are solely derived from the SEER database and lack relevant information on patients from other medical regions. Secondly, certain imaging-related data that may contribute to the prediction of lymph nodes are lacking in the SEER database. We hope that further research will address these limitations.

## Conclusion

In this study, we developed a nomogram for predicting lymph node metastasis (LNM) in CRC patients. We identified tumor location, grade, age, tumor size, T stage, race, and CEA as independent predictive factors for LNM in CRC patients. This tool can predict the likelihood of LNM in CRC patients, which may aid clinicians in formulating appropriate treatment strategies.

## Data Availability

The data for this study are publicly available from the Surveillance, Epidemiology, and End Results database (https://seer.cancer.gov/).
